# Non-surgical management of recurrent perforation of a jejunal diverticulum following previous segmental bowel resection: a case report

**DOI:** 10.4076/1752-1947-3-7318

**Published:** 2009-07-14

**Authors:** Hugh Shunsuke Colvin, Chin Kuenfoo, Taufiek Konrad Rajab, Thomas Sayadatas

**Affiliations:** 1 Addenbrookes Hospital, Cambridge University Hospitals NHS Foundation Trust, Hills Rd, Cambridge, CB2 0QQ, UK; 2 Imperial College London, Exhibition Road, London SW7 2DD, UK; 3 Universitaetsfrauenklinik, University of Tuebingen, Calwerstr 7, Tuebingen, Germany

## Abstract

**Introduction:**

Perforations of jejunal diverticula are uncommon and repeated symptomatic perforations have been reported only twice before in the literature. This is the first case report of recurrent perforation of a jejunal diverticulum to be successfully managed non-operatively.

**Case presentation:**

We report a recurrent perforation of a jejunal diverticulum in an 87-year-old Caucasian man who presented with a 1-week history of epigastric pain. The diagnosis of a perforated jejunal diverticulum was made from the appearances of the abdominal computed tomography scan together with the presence of jejunal diverticula noted at the time of a previous laparotomy for the first perforation of a jejunal diverticulum.

**Conclusion:**

Whilst this case report by itself does not add to the knowledge we already have of jejunal diverticula, it is one report of a rare condition and more reports are required in the future to establish the recurrence rate of jejunal diverticula perforation and how perforated jejunal diverticula are best managed.

## Introduction

This is a rare case of repeated perforations of jejunal diverticula. To the best of our knowledge, there are only two previous similar reports [1,2]. This is however the first reported case of a recurrent perforation being treated successfully non-operatively.

Jejunal diverticula are uncommon, with reported incidences at autopsy of 0.26% to 1.3%, occurring mostly after the sixth decade of life and slightly more often in men than in women. Jejunal diverticula occur on the mesenteric side of the bowel wall, which is weakened by the penetration of blood vessels [3]. They are thought to be acquired pulsion diverticula, arising as a result of motor dysfunction of the smooth muscle or the myenteric plexus in the small bowel [4,5]. The diverticula are usually distributed in the proximal jejunum [6], but they can be present anywhere along the small bowel and can be extensive in their distribution [7].

A significant proportion of people with jejunal diverticula have chronic symptoms including abdominal pain after food associated with nausea, vomiting, belching, flatulence, diarrhoea, or constipation [8]. Serious and acute complications arise less frequently and include gastrointestinal haemorrhage, perforation and intestinal obstruction, occurring in 6% to 10% of patients with jejunal diverticula [5,7,9]. Perforations occur in 2.3 to 6.4% of people with jejunal diverticula and in the majority of cases, they arise from acute necrotising inflammation, whilst blunt trauma or foreign bodies can also precipitate perforations [4,10].

## Case presentation

**Figure 1:**
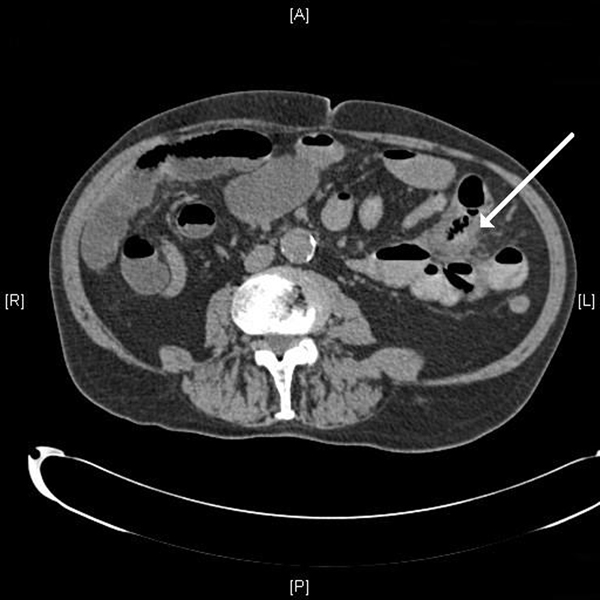
**Computed tomography scan demonstrating marked soft tissue stranding containing multiple small locules of gas and which is surrounded by several small bowel loops (arrow)**. This area appears to connect to a loop of small bowel but does not definitely lie in continuity with the rest of the bowel, which is very suspicious for a localised perforation of the small bowel.

An 87-year-old man presented with a 1-week history of episodic, sharp and non-radiating epigastric pain. He also had abdominal bloating, anorexia, vomiting and loose stools. Three years previously, he had a laparotomy for perforated jejunal diverticula that required segmental jejunal resection and primary end-to-end anastomosis. The resection was of a 45 cm length of jejunum that contained two inflamed and perforated diverticula but the adjacent and distal 45 cm segment of jejunum containing non-inflamed diverticula was left alone. The man's other background history was of colonic diverticula disease, right inguinal hernia repair and transurethral resection of the prostate. On examination, his abdomen was distended with tenderness and guarding in the right upper quadrant but without overt peritonism as he had a soft abdomen without rebound tenderness. His pulse was regular at 117 beats per minute, blood pressure was 130/90 mmHg, and he was apyrexial. His C-reactive protein level was raised at 46 mg/L whilst his white blood cell count was within the normal limits at 7.7 × 10^9^/L. His renal and liver function tests were normal. Erect chest X-ray did not reveal free air under the diaphragm. A computed tomography (CT) scan of the abdomen showed evidence of a localised perforation of the small bowel with multiple dilated loops of small bowel surrounding an area of marked soft tissue stranding with multiple small locules of gas, which appeared to connect to a loop of small bowel (Figure 1). The CT finding, together with the presence of jejunal diverticula, was most consistent with the diagnosis of a second perforation of the jejunal diverticula.

It was felt most appropriate to treat the man conservatively as he remained stable and without features of peritonism, and so a nasogastric (NG) tube was inserted to decompress the small bowel together with administration of intravenous fluids, ciprofloxacin and metronidazole. He made a good symptomatic recovery and was discharged on the fourth day after admission on a course of oral antibiotics and has remained well since.

## Discussion

Perforated jejunal diverticula are managed according to their presentation and the fitness of the patient. Peritonitis caused by perforated jejunal diverticula can be localised and self-limiting because the diverticula are at the mesenteric border of the bowel and readily allow the small bowel mesentery to wall them off [5,7]. If the perforation of a jejunal diverticulum causes only localised peritonitis and the patient remains stable, it is the view of Novak *et al.* that a trial of non-surgical management with intravenous antibiotics and other supportive measures alongside percutaneous CT-guided aspiration of localised intraperitoneal collections may be suitable and avoid the need for surgery [11]. Non-surgical management of perforated jejunal diverticula is a relatively new idea and the evidence for it is limited to case reports such as the current one that demonstrates that a good outcome can be obtained [11]. In the current case, the 1-week history of abdominal pain during which the patient remained stable favoured the conservative approach.

Clinicians must also be wary of the mortality rate from perforated jejunal diverticula which can be as high as 21% to 40% according to some reports, particularly in those who are old or have a delayed diagnosis [4]. Other complications resulting from perforation of jejunal diverticula include the formation of fistulas between small bowel segments, colon and urinary bladder, suppurative pyelophlebitis and multiple hepatic abscesses, and abdominal wall abscess [5]. Therefore, the current treatment of choice for perforated jejunal diverticula that causes generalised peritonitis or compromises the patient's condition is prompt laparotomy with segmental intestinal resection and primary anastomosis [12]. The extent of the bowel resection depends upon the length of the bowel which is affected by the diverticula and the patient's perioperative condition [10]. If diverticula are extensive, which is quite commonly the case [10,12], resection may have to be limited to include only the segment containing the perforated diverticulum and to leave a segment of small bowel that still contains non-perforated diverticula in order to avoid short bowel syndrome [1]. The presence of the retained diverticula must be recorded for future reference to aid rapid recognition of any complications arising from them. However, even when the entire length of the small bowel affected by diverticula is resected, diverticula can recur in a different segment of the small bowel which was unaffected by them at the time of the operation [6].

Other surgical techniques used in the past for the treatment of perforated jejunal diverticula were to invaginate the diverticulum with a suture, or to suture the perforation and then cover it with an omental patch [10]. Both of these older techniques are a challenge to perform well on jejunal diverticula, which are positioned rather inaccessibly next to the mesentery and have fallen out of favour because they are associated with three times the mortality rate compared with segmental bowel resection and primary anastomosis [4,5,10].

A promising alternative to conventional laparotomy is to use the laparoscope to diagnose the perforated jejunal diverticula as well as to guide the surgeon to the ideal incision site on the abdominal wall to allow the segmental bowel resection and primary anastomosis to be carried out through a smaller and better positioned cut. A smaller incision leads to less postoperative pain and morbidity [13].

Whilst there are other reports of 'micro-perforations' of jejunal diverticula leading to chronic asymptomatic pneumoperitoneum [14], there are only two previous reports of recurrent and symptomatic perforation of jejunal diverticula in the same patient. In 1982, Alvarez *et al.* reported a 74-year-old man who presented with peritonitis from perforated jejunal diverticula on two occasions separated by 2 years, and in 2002, Franzen *et al.* reported a 69-year-old man who also presented with peritonitis from perforated jejunal diverticula on two occasions separated by 13 weeks. In each of the episodes of jejunal diverticula perforation in the above two cases, segmental bowel resection and primary anastomosis were carried out with good effect [1,2]. Franzen *et al.*'s case is similar to our case in that not all of the jejunum affected by diverticula was resected in the initial operation, whilst we are not sure if the case reported by Alvarez *et al.* arose from a perforation of a jejunal diverticulum that had already been present at the time of the initial surgery or from one that had developed since.

The combination of the low incidence of jejunal diverticula amongst the population as well as the low rate of perforation of jejunal diverticula [3,4,10] would make repeated perforations of jejunal diverticula rare, but there may be additional explanations for their rarity. Perforation of jejunal diverticula often prompts bowel resection which may also involve the removal of coexisting jejunal diverticula thereby eliminating their chances of perforating in the future. There may also be an underreporting of perforated jejunal diverticula by clinicians due to it being frequently misdiagnosed [11], or even by patients themselves who do not decide that their symptoms are severe enough to warrant a visit to the doctor in cases where the peritonitis is mild, localised and self-limiting [5,7]. Indeed, it was only after a duration of one week from the onset of pain that our patient decided his symptoms required medical attention.

## Conclusion

Whilst this case report by itself does not add to the knowledge we already have of jejunal diverticula, it is a report of a rare condition and more reports are required in the future to establish the recurrence rate of jejunal diverticula perforation and how perforated jejunal diverticula are best managed.

## Consent

Written informed consent was obtained from the patient for the publication of this case report and any accompanying images. A copy of the written consent is available for review by the Editor-in-Chief of this journal.

## Competing interests

The authors declare that they have no competing interests.

## Authors' contributions

HSC performed the literature review and drafted the manuscript. CK performed the literature review and drafted the manuscript. TKR revised the manuscript critically for important intellectual content. TS revised the manuscript critically for important intellectual content.
